# Surgical Aid Society

**Published:** 1897-04-10

**Authors:** 


### SURGICAL AID SOCIETY.

It must be satisfactory to dwellers in the country to know that the Surgical Aid Society is establishing local branches, so that the advantages of surgical aid may be brought to people in the provinces. A case has been brought to our notice in the county of Kent of the wife of a policeman who was troubled with her teeth, and who has been able to secure much-needed relief through the Tunbridge Wells branch of the Surgical Aid Society. Having regard to the fact that the Hospital Sunday and Hospital Saturday Funds now provide adequate surgical appliances and surgical aid upon a most excellent plan, without tickets, for poorer Londoners, we think the Surgical Aid Society might usefully extend its district branches and so secure not only a much greater volume of support for itself, but do an incalculable amount of good by enabling poor persons who at present are without necessary surgical appliances to secure all they need promptly and readily.

